# Cancer Microenvironment Defines Tumor-Infiltrating Lymphocyte Density and Tertiary Lymphoid Structure Formation in Laryngeal Cancer

**DOI:** 10.1007/s12105-022-01517-7

**Published:** 2022-12-31

**Authors:** Anastasia G. Gkegka, Michael I. Koukourakis, Michael Katotomichelakis, Alexandra Giatromanolaki

**Affiliations:** 1 Department of Pathology, Democritus University of Thrace, University Hospital of Alexandroupolis, Alexandroupolis, Greece; 2 Department of Radiotherapy / Oncology, Democritus University of Thrace, University Hospital of Alexandroupolis, Alexandroupolis, Greece; 3 Otolaryngology - Head and Neck Surgery, Democritus University of Thrace-General, University Hospital of Alexandroupolis, Alexandroupolis, Greece

**Keywords:** Laryngeal cancer, Tumor-infiltrating lymphocytes, HIF1α, LDH, Angiogenesis

## Abstract

**Background:**

The presence and activity of tumor-infiltrating lymphocytes (TILs) is a key parameter related to the antitumor immune response. A large number of studies reveal TIL density as a prognostic marker and predictor of response to radiotherapy, chemotherapy, and immunotherapy.

**Methods:**

We examined the TIL and tertiary lymphoid structure TLS density in the invading front and inner tumor stroma, in a 33 squamous cell laryngeal carcinomas (LSCC) treated with laryngectomy. TIL and TLS densities were in parallel examined with markers of anaerobic metabolism, vascular density (VD), vascular survival ability (VSA), and histopathological parameters.

**Results:**

TIL and TLS densities significantly decreased in inner tumor areas (*p* < 0.0001). TIL density in the invading tumor front was inversely related with lymph node involvement (*p* = 0.03), HIF1α expression (*p* = 0.008), vessel density (*p* = 0.02), and MIB1 (*p* = 0.006). TIL density in inner stroma was inversely linked to local invasion (marginal *p* = 0.05), tumor budding (TB) (*p* = 0.005), MIB1 (*p* = 0.02), and HIF1α expression (*p* = 0.02). Low-TLS density in the invading front and in inner tumor areas was related to high TB (*p* = 0.02 and 0.002, respectively), HIF1α (*p* = 0.003 and 0.01, respectively), and LDH5 expression (*p* = 0.003 and 0.007, respectively). CD4^+^, FOXP3^+^ TIL density, and FOXP3^+^/CD8^+^ ratio were directly associated with VSA (*p* = 0.008, 0.02, and 0.05, respectively).

**Conclusion:**

Poor immune response is related to hypoxic background and anaerobic metabolism, as well as increased invasive and metastatic ability. Regulatory TIL markers are linked with increased angiogenic potential. The prognostic, predictive, and therapy-guiding value of TILs in clinical practice demands thorough investigation.

**Supplementary Information:**

The online version contains supplementary material available at 10.1007/s12105-022-01517-7.

## Introduction

Squamous cell laryngeal carcinoma (SCC) is the second most commonly diagnosed malignancy among head and neck (HN) tumors in the West, associated etiologically with the use of tobacco and alcohol [1–3]. The mortality of laryngeal SCC is high, as in 2017, 210.606 people developed SCC of the larynx in the world, and 126.471 passed away [4]. Although hemi-laryngectomy and radiotherapy (RT) provide excellent control of the disease in early stages, the cure rates for locally advanced laryngeal SCC, treated with total laryngectomy and postoperative RT or radical chemo-RT, are below 50%. Metastatic disease is treated with chemotherapy and palliative radiotherapy [5]. Immunotherapy has stayed on the frontline the recent years. The scientific community is more and more studying the immune response to HN-SCCs, anticipating the discovery of more effective and specific immune checkpoint inhibitors. So far, FDA has approved anti-PD-1 antibodies, pembrolizumab, and nivolumab, as immunotherapeutic agents for platinum-refractory HN-SCC [6].

In 1863, Rudolph Virchow introduced to the scientific community the importance of the tumor microenvironment (TME). Virchow focused on the immune cells of the TME [7], and it was not before 1889, since Paget referred to all the other cells and molecules that are present [8]. Pending on the type of their activation and metabolism, the non-cancer tumor component (fibroblasts, immune, and endothelial cells) has a critical role suppressing or promoting tumor growth, invasion, and metastasis. Thus, the close interplay between cancer cells and TME defines patient’s prognosis [9, 10].

Tumor-infiltrating lymphocytes (TILs) are known to affect the prognosis of HN cancer patients [11], as well as the response to RT and chemotherapy [12]. As a tumor grows in a microenvironment with abnormal dysfunctional vasculature and poor oxygen availability, cancer cells use anaerobic glycolysis to cover their metabolism needs. Hypoxic and acidic conditions prevail in the TME. Moreover, for oncogenic or other reasons, up-regulation of Hypoxia-Inducible Factors and the AKT pathway, cancer cells prefer to use glycolysis and anaerobic metabolic pathways regardless of the presence of oxygen, a phenomenon known as “Warburg effect” [13]. Last but not least, while cancer cells need oxygen and nutrients to thrive, the secretion of angiogenic factors, like VEGF, triggers angiogenesis on the frontline of the invading tumor mass. All these processes and conditions can be studied at the pathology level using microscopy and immunohistochemistry. Accumulations of lymphocytes and formation of lymphoid structures (with or without a germinal center), tertiary lymphoid structures (TLSs), caused by cancer/host tissue interactions, are also easy to notice in H&E. Immunohistochemistry for specific CD markers can be used to quantify the density of TIL subtypes with effector or regulatory activity.

In the present study, we examined the TIL density and TLS density in the tumor periphery and inner tumor areas, in a series of laryngectomy surgical samples from patients with laryngeal SCCs. The obtained values were studied in relation to markers of hypoxia, angiogenesis, and histopathological parameters, including cancer cell proliferation, p16, and SMA expression.

## Materials and Methods

Surgical samples from thirty-three patients treated with laryngectomy for SCC of the larynx were included in the study. The retrieved tissue blocks were consecutive according to the date of their registration in the archives of our department. The study has been approved by the local Ethics and Research Committee of the University Hospital of Alexandroupolis (ES11/26-11-2018).

The histopathological parameters taken into account for the current analysis were the tumor size (in cm), the invasiveness of the tumor to soft tissue vs. cartilage, and the involvement of regional lymph nodes (negative vs. positive).

### Assessment of TILs

TIL assessment was carried out in hematoxylin and eosin-stained slides. Assessment was performed in one tissue section selected among the sections cut from all available paraffin blocks. The selection of the section was based upon the presence of a clear invading tumor front, adequate overall material with clear stroma, and lack of extensive necrosis. All high-power × 40 fields were examined in the whole slide, and TILs were calculated separately in the invading front and the inner stroma of the tumor. In the microscope used for the evaluation, one high-power × 40 field (10 × eyepiece and 40 × objective − 400 × magnification) corresponds to 0.25 mm2. Areas of necrosis or ulceration were ruled out. The total number of lymphocytes was divided by the overall number of optical fields (o.f.) on the corresponding slide to provide the final score of each case (mean value of all fields) in the tumor periphery and in inner areas. The median value of the obtained scores was used to group tumors in two categories of low- and high-TIL density.

As ‘invading front’ (or ‘tumor periphery’) was defined the area comprised by the first × 400 optical field containing tumor-invading front and normal tissue adjacent to this front (50% of normal and 50% of cancer). TLSs were identified in the stroma of the invading tumor and in the normal tissue infiltrated by the tumor. Inner tumor areas refer to all available × 40 optical fields of the tumoral tissue section immediately below the optical field that defined the tumor periphery. TLSs were identified in the stroma areas (see Supplemental Fig. 1s).

### Assessment of TLSs

All tertiary lymphoid structures with or without identifiable germinal centers were recorded. TLS evaluation was performed in hematoxylin and eosin-stained slides. The number of TLSs was assessed in all high-power × 40 fields in the invading front of the tumor and inside the tumor stroma. Areas of necrosis or ulceration were excluded. The sum of all TLSs was divided by the entire number of optical fields (o.f.) on the analogous slide, and a final score was obtained for each case (mean value of all fields). Tumors were categorized in two groups of low- and high-TLS density according to the median value of the acquired scores.

### Assessment of Tumor Budding (TB)

Tumor budding (TB) was evaluated according to the recommendations for reporting tumor budding that were proposed in International Tumor Budding Consensus Conference (ITBCC) 2016 [14]. Tumor budding was considered as any cluster of one to four neoplastic cells in the invading front of the tumor, that had no connection with it. The whole tissue slide was scanned in a low-power field (magnification, × 40 and × 100), the area with the higher density in TB (hot-spot) was chosen, and the tumor buds were calculated in one × 200 optical field. One TB value corresponded to each tumor. Using the median value (≤ median vs. > median), cases were grouped into low and high TB groups.

### Immunohistochemistry

Immunohistochemistry for markers (except LDH5) was performed as follows: Sections, 3 μm thick, were deparaffinized and placed in antigen-retrieval target solution pH 9.0 (DAKO), followed by microwaving (3 × 5 min). The primary monoclonal antibodies were applied. Following washing with phosphate-buffered saline (PBS), endogenous peroxidase was quenched with EnVision Flex Peroxidase Block (DAKO) for 10 min, then sections were washed with PBS. Non-specific binding was blocked in EnVision Flex mouse Linker for 15 min (DAKO), and then sections were washed with PBS. Sections were then incubated with a secondary antibody (EnVision Flex/HRP; DAKO) for 30 min, and washed in PBS. The color was developed by 5-min incubation with 3,3’-diaminobenzidine (DAB) solution (for CD31 marker was also used HRP Magenta Substrate Chromogen System/DAKO) and sections were counterstained weakly with hematoxylin.

The primary antibodies used are described along the following lines:

- SMA: Mouse monoclonal, 1A4 (DAKO), dilution 1:50, 60-min incubation
- Ki-67: Mouse monoclonal, MIB1 (DAKO), dilution 1:60, 60-min incubation
- p16: Mouse monoclonal, IHC016 (GenomeMe), dilution 1:100, 60-min incubation
- HIF1α: Mouse monoclonal, ESEE122 (OXFORD), dilution 1:10, overnight incubation
- CD4: Mouse monoclonal, 4B12 (DAKO), dilution 1:40, 60-min incubation
- CD8: Mouse monoclonal, C8/144B (DAKO), ready to use, 60-min incubation
- FOXP3: Mouse monoclonal, 236A/37 (OXFORD), dilution 1:50, overnight incubation

A different procedure was followed for LDH5 immunohistochemical marker: Sections, 3 μm thick, were deparaffinized and placed in an antigen-retrieval target solution pH 9.0 (DAKO), followed by microwaving (3 × 5 min). Non-specific binding was blocked in normal rabbit serum at a dilution of 1:20 for 30 min (DAKO, X0902). No rinsing was performed. The primary Polyclonal antibody to isoenzyme LDH5 was applied at a dilution of 1:200 overnight at 4 °C. Following washing with PBS, endogenous peroxidase was quenched with EnVision Flex Peroxidase Block (DAKO) for 10 min. It was followed by washing with PBS. Sections were, then, incubated with a secondary antibody (Biotinylated Secondary rabbit-antisheep; DAKO, P0163) at a dilution of 1:100 for 30 min, and washed in PBS. Streptavidin-HRP (DAKO) at dilution of 1:100 was applied for 30 min, and sections were again washed in PBS. The color was developed by 15-min incubation with DAB solution and sections were counterstained weakly with hematoxylin [15].

### Assessment of SMA, p16, and MIB1 Proliferation Index

SMA immunohistochemical marker was used for the evaluation of cancer-associated fibroblasts-(CAFs) density. Vessels were used as an internal control (and were excluded from the assessment). The percentage of positive SMA stroma expression was estimated in all × 200 power fields and the mean value was obtained to score each case. In accordance with the median value of these cores, tumors were divided in three categories: low < 5%, medium 5–50%, and high > 50%.

Evaluation of p16 was performed in all high-power × 400 optical fields of the slide, and their mean value was used to score each case. Positive for p16 marker was considered strong and diffuse cytoplasmic and nuclear staining. Given the low expression of p16 in laryngeal cancer, assessment of the % of p16 expressing cells was performed in higher magnification to assure a more precise quantification.

MIB1 nuclear expression was assessed in × 200 optical fields of the tumor in the whole tissue slide. The percentage of cells with nuclear MIB1 expression was recorded per field and their mean value was used to provide a score for each case.

### Assessment of Hypoxia-Inducible Factor-1α and Lactate Dehydrogenase

The patterns of expression of HIF1α and LDH5 that were considered for analysis were strong cytoplasmic and nuclear, as previously reported [16]. The percentage of cancer cells presenting these patterns was recorded in all × 200 optical fields and the mean value was used to score each case. Weak cytoplasmic expression was considered as negative. Cases were divided in two groups of low vs. high HIF1α or LDH5 expression. High expression was defined when cytoplasmic strong expression was above the 50% of cancer cells and/or when nuclear expression was higher than 10%.

### Assessment of Vascular Density (VD) and Vascular Survival Ability (VSA)

Calculation of the number of microvessels was used to evaluate angiogenesis. Each tumor slide was, at first, scanned at low-power fields (magnification, × 40 and × 100). The area with the highest vascular density in the invading front of the tumor was chosen and CD31 immunohistochemistry was used for the assessment in × 200 optical field. Moving toward the tumor core, the immediately adjacent two × 200 optical fields were assessed. Thus, a total of three fields (t1, t2, and t3) were included in the assessment, from the periphery to the center of the tumor, and three mean values were obtained for each case. Using the ratio of VD t2/t1 and t3/t1, we calculated the Vascular Survival Ability (VSA) of each tumor. This index shows the ability of vessels to survive in inner tumor area. The method has been extensively validated in the previous studies of ours [17].

### Assessment of CD4^+^, CD8^+^, and FOXP3^+^ TIL Density

All three antibodies for lymphocytes were evaluated in the same way. The percentage of CD4^+^, CD8^+^, and FOXP3^+^ positive lymphocytes was estimated in all high-power × 400 optical fields, separately in the invading front, and the inner stroma of the tumor. A mean value of all fields was obtained for each marker on each case. The regulatory to effector T-cell CD4^+^/CD8^+^ and FOXP3^+^/CD8^+^ (regulatory/effector) ratios were, thereafter, calculated.

### Statistical Analysis

Statistical analysis and the creation of graphs was performed using the GraphPad Prism 7.0 package. The non-parametric Mann–Whitney test (for two variables) or the Kruskal–Wallis non-parametric test with subsequent Dunn test for intergroup comparison (for multiple variables) was used to compare categorical continuous tumor variables. The Wilcoxon matched-pairs signed rank test was used to compare continuous paired variables. A *p* value < 0.05 was used for significance.

## Results

### TIL and TLS Densities

**Fig. 1:**
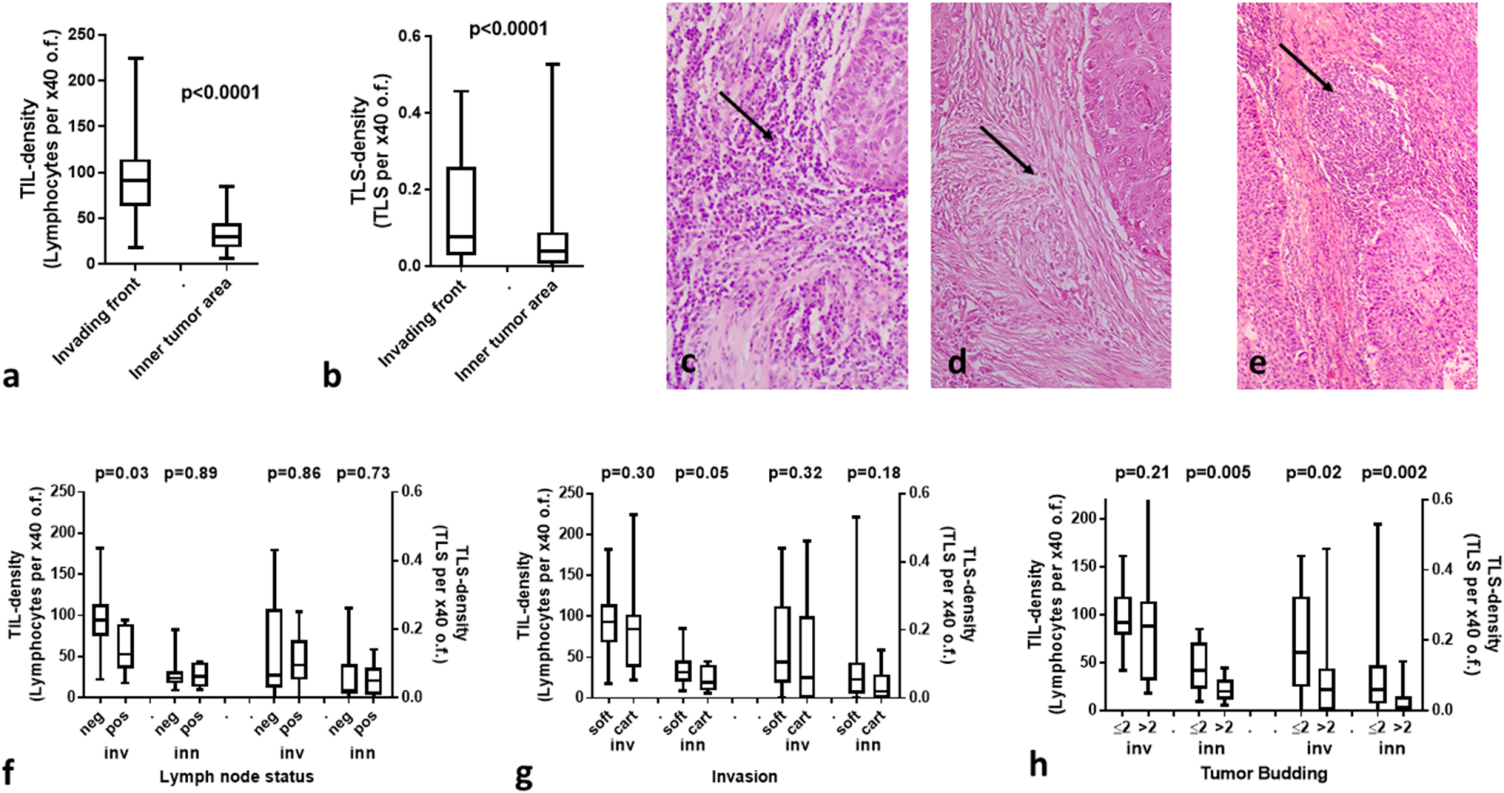
TIL and TLS densities and correlations with histopathological variables: **a** TIL density in the invading tumor front vs. inner tumor areas, **b** TLS density in the invading tumor front vs. inner tumor areas, **c** a typical image from H&E stained tissue section of a laryngeal SCC showing dense infiltration by TILs in the invading tumor front (magnification × 200), **d** low-TIL density in the invading front of a laryngeal SCC (magnification × 200), **e** a typical image of a TLS in the invading tumor front (magnification × 200), **f** TIL and TLS densities in the invading front and inner tumor areas in tumors with and without lymph node involvement, **g** TIL and TLS densities in the invading front and inner tumor areas, in tumors with and without invasion of the laryngeal cartilage, **h** TIL and TLS densities in the invading front and inner tumor areas, in tumors with low and high tumor budding

The median TIL density in the tumor periphery was 91 (18–224), while in inner tumor areas it dropped to 51 (6–85) (*p* < 0.0001; Fig. 1a). The median TLS density (TLS per o.f.) in the tumor periphery was 0.07 (0.02–0.45) and in inner tumor areas 0.03 (0.007–0.52) (p < 0.0001; Fig. 1b). Figure 1c, d shows typical examples of dense and poor TIL infiltration, and Fig. 1e shows TLS presence, in the invading tumor front.

### Histopathological Variables vs. TILs/TLSs

The tumor size ranged from 1.2–8 cm (median 3 cm). Using the median values, cases were split in two groups of small vs. large tumors (≤ median vs. > median). Analysis of TIL density and TLS density, in the invading front (inv) and inner tumor areas (inn), did not show any statistically significant association with tumor size (*p* > 0.35).

The invasiveness of laryngeal tumors was grouped in two categories; tumor that invades soft tissue vs. tumors that invade the cartilage or the hyoid bone. Figure 1f shows the distribution of TIL and TLS densities. Low-TIL density in inner tumor areas was marginally linked with invasion to cartilage structures (median 19 vs. 31; *p* = 0.05).

Analysis of TILs and TLSs according to the presence of lymph node metastatic involvement showed that low-TIL density in the invading tumor front was significantly related to higher metastatic potential (median TIL density 52 vs. 94; *p* = 0.03; Fig. 1g).

Tumor budding (TB) counting in the hot-spot showed a range of 0–19 TBs, median 2. High TB numbers were linked with low-TIL density in inner tumor areas (*p* = 0.005), low-TLS density in the invading tumor front (*p* = 0.02), and low-TLS density in inner tumor areas (*p* = 0.002; Fig. 1h).

SMA expression was grouped in low/medium vs. high. TIL and TLS densities in the invading tumor front and inner tumor areas were analyzed according to SMA groups. There was no significant difference (*p* > 0.19).

Regarding p16, only 10/33 tumors showed reactivity in a very low percentage of cancer cells (0–4%), so further analysis was not feasible.

**Fig. 2:**
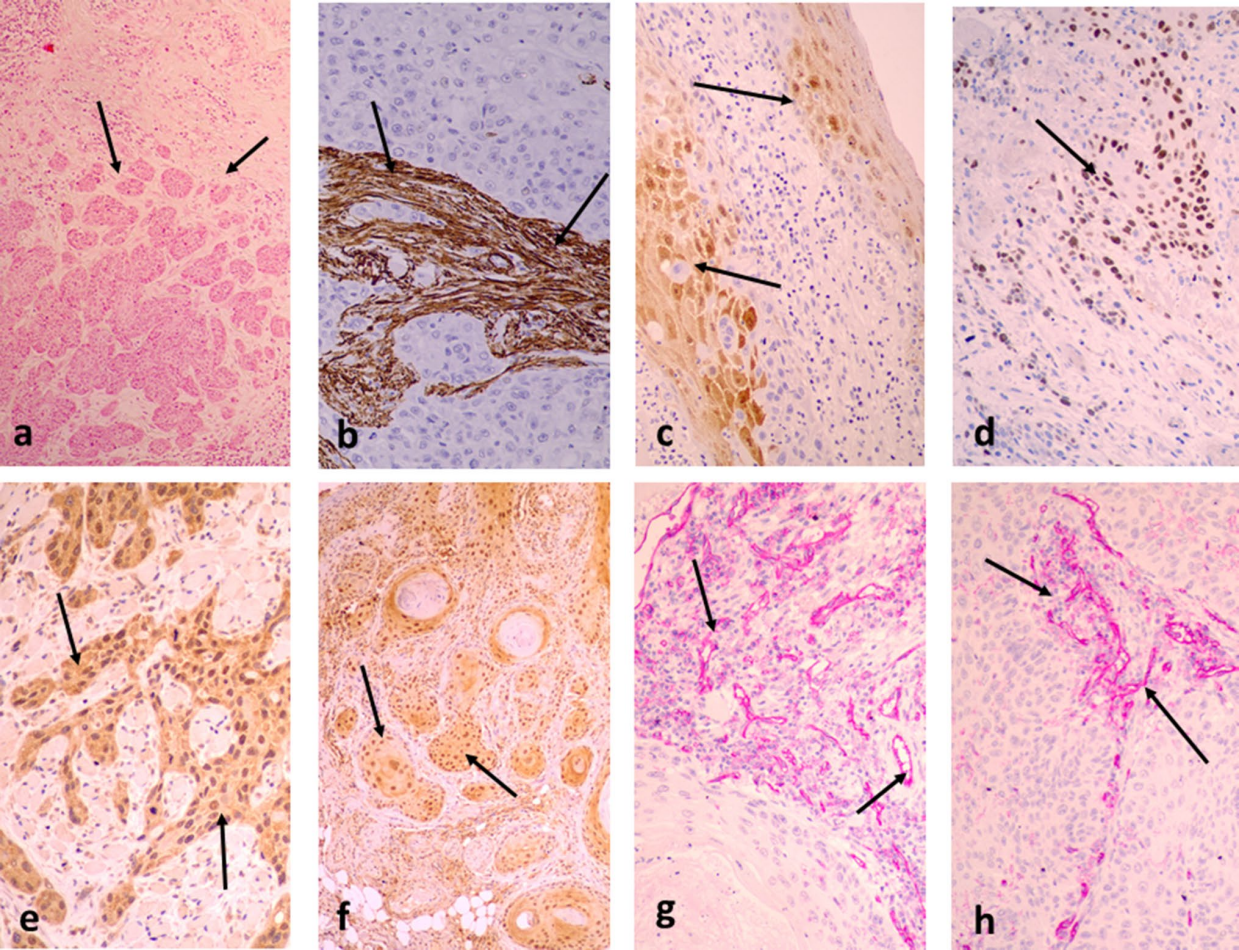
Typical immunohistochemical images: **a** high tumor budding in the invading tumor front of a laryngeal carcinoma (arrows), **b** intense SMA staining of the tumor stroma (arrows), **c** p16 cytoplasmic and nuclear protein expression (arrows), **d** nuclear expression of MIB1 in cancer cells (arrows), **e** nuclear and cytoplasmic HIF1α expression (arrows), **f** nuclear and cytoplasmic LDH5 expression (arrows), **g** dense CD31^+^ vascular density in the invading tumor front (arrows), **h** dense CD31^+^ vascular density in inner tumor areas (arrows)

High MIB1 proliferation index was associated with poor TIL density in the invading tumor front (p = 0.006, r = 0.47) and in inner tumor areas (*p* = 0.02, *r* = 0.40). Figure 2 shows typical immunohistochemical images of TB, SMA, p16, and MIB1 expression.

### HIF1α/LDH5 vs. TILs/TLSs

**Fig. 3:**
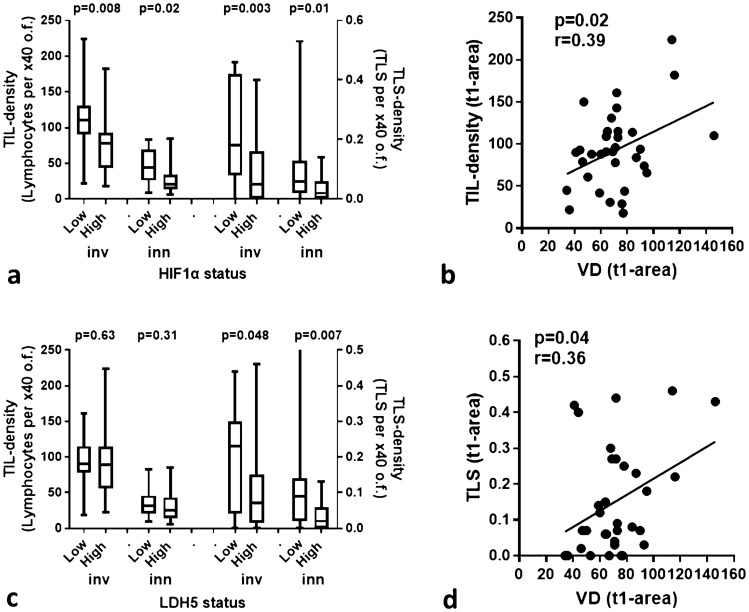
TIL and TLS density associations with HIF1α, LDH5, and vascular density: **a** TIL and TLS densities in the invading front and inner tumor areas, in tumors with low and high HIF1α expression, **b** TIL and TLS densities in the invading front and inner tumor areas, in tumors with low and high LDH5 expression, **c** linear regression analysis of vascular density (VD) in the invading tumor front (t1 area) and TIL density in t1 area, **d** linear regression analysis of vascular density (VD) in the invading tumor front (t1 area) and TLS density in t1 area

Figure 2 shows typical immunohistochemical images of HIF1α and LDH5 expression. Cytoplasmic HIF1α expression ranged from 0 to 95% (median 41), while nuclear expression ranged from 0 to 60% (median 0%) of cancer cells. High expression of HIF1α was noted in 18/33 laryngeal tumors. This was linked with poor TIL density in the tumor-invading front and in inner tumor areas (*p* = 0.008 and *p* = 0.02, respectively; Fig. 3a). In addition, high HIF1α was linked with poor TLS density in the tumor-invading front and in inner tumor areas (*p* = 0.003 and *p* = 0.01, respectively; Fig. 3a).

Cytoplasmic LDH5 expression ranged from 0 to 66% (median 13), while nuclear expression ranged from 0 to 60% (median 12%) of cancer cells. High expression of LDH5 was noted in 18/33 laryngeal tumors. High expression of LDH5 was linked with poor TLS density in the tumor-invading front and in inner tumor areas (*p* = 0.048 and *p* = 0.007, respectively; Fig. 3b).

### VD and VSA vs. TILs/TLSs

Figure 2 shows typical immunohistochemical images of high VD in the invading front and inner tumor areas. The vascular density VD in the invading tumor front ranged from 23 to 146 microvessels per × 200 optical field (median 71). The median VD dropped to 40 and 36 in t2 and t3 inner tumor areas; *p* < 0.0001. Using the ratio of VD t2/t1 and t3/t1, we calculated the Vascular Survival Ability VSA of each tumor, which ranged from 0.19 to 1 for t2/t1 and from 0.05 to 1 for t3/t1. Linear regression analysis of the above angiogenesis-related variables with the TIL and TLS densities showed that VD in the invading tumor front (t1 area) was directly related to TIL density (*p* = 0.02, *r* = 0.39) and TLS density (*p* = 0.04, *r* = 0.36) in the invading tumor front; Fig. 3c and d, respectively. No other significant associations were noted.

### TIL Subtypes and Histopathological Variables

**Fig. 4:**
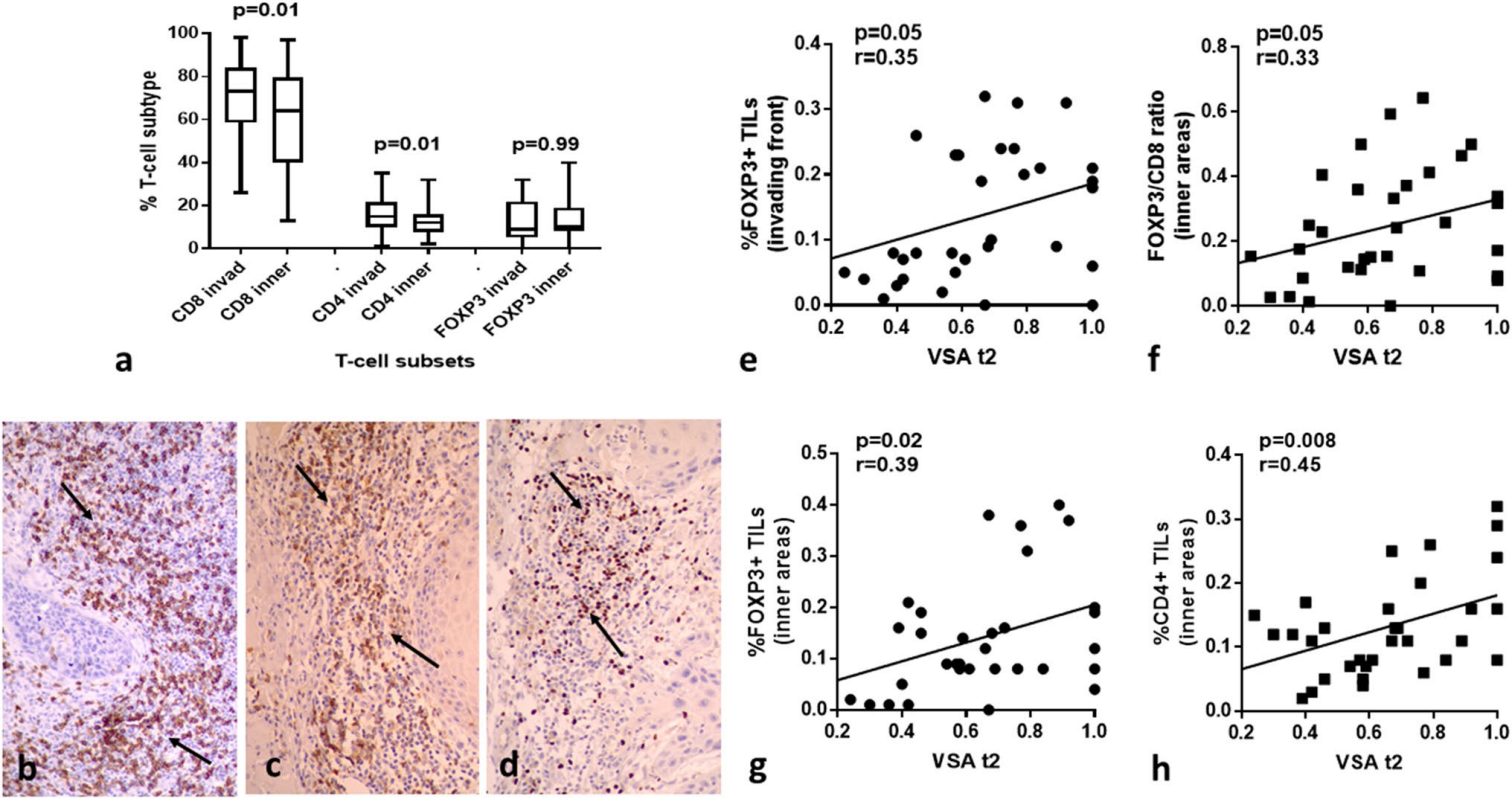
T-cell subtypes, their density, and associations with vascular survival ability: **a** CD8^+^, CD4^+^, and FOXP3^+^ TIL density in the invading tumor front and inner tumor areas, **b** intense presence of CD8 ^+^ T-cells infiltrating the invading tumor front, **c** intense presence of CD4 ^+^T-cells infiltrating the invading tumor front, **d** intense presence of FOXP3^+^ T-cells infiltrating the invading tumor front, **e** linear regression analysis of vascular survival ability (VSA, assessed by comparison of t1/t2 areas) and FOXP3^+^ TIL density in the invading tumor front, **f** linear regression analysis of vascular survival ability (VSA) and FOXP3^+^/CD8 ^+^ in inner tumor areas, **g** linear regression analysis of vascular survival ability (VSA) and FOXP3^+^ TIL density in inner tumor areas, **h** linear regression analysis of vascular survival ability (VSA) and CD4 ^+^ TIL density in inner tumor areas

Figure 4a shows the % of CD8^+^, CD4^+^, and FOXP3^+^ TILs counted in the invading tumor front and in inner tumor areas. CD8^+^ and CD4^+^ TILs decreased in inner tumor areas (*p* = 0.01). We further calculated the CD4^+^/CD8^+^ and FOXP3^+^/CD8^+^ (regulatory/effector) ratios, but there was no significant difference between the invading front and inner tumor areas (*p* > 0.07). Figures 4b–d show typical immunohistochemical images of dense CD8^+^, CD4^+^, and FOXP3^+^ lymphocytic infiltration.

Analysis of the % of CD8^+^, CD4^+^, FOXP3^+^ TIL subtypes and CD4^+^/CD8^+^, FOXP3^+^/CD8^+^ ratios (as assessed in the invading tumor front) between the groups of tumors with and without invasion to laryngeal cartilage showed no significant differences (data not shown). A marginally lower presence of CD8^+^ TILs in tumors with cartilage invasion was noted (*p* = 0.07). Similar analysis of the TIL subsets in inner tumor areas did not show any association with invasion of laryngeal cartilage. A similar analysis did not reveal any association with tumor size, SMA expression, and tumor budding.

### TIL Subtypes and Microenvironmental Factors

Using linear regression analysis, we assessed the association of the above-mentioned TIL qualities with the expression of HIF1α, LDH5, VD (t1, t2, and t3), and VSA (t2 and t3) in the invading tumor front (Supplemental Table 1). Direct association of FOXP3^+^ TILs with VSA t2 was noted (marginal significance *p* = 0.05; Fig. 4e). In inner tumor areas a direct association of CD4^+^, FOXP3^+^ TILs, and FOXP3^+^/CD8^+^ ratio with VSA t2 was noted (*p* = 0.008, 0.02, and 0.05, respectively; Fig. 4f–h).

## Discussion

Cancer microenvironment has been widely studied in the last decades, as it plays a crucial role in cancer progression. Tumor stroma directly interacts with cancer cells, most often to function as an accomplice for tumor growth and invasion [18]. Immune cells in the tumor microenvironment act in such a way to either attack cancer cells or suppress immune interactions to allow tumor progression. Many studies support the importance of quantitative and qualitative characteristics of the immune infiltrate in the prognosis of patients. In particular, tumor-infiltrating lymphocytes (TILs) and tertiary lymphoid structures (TLSs) are found to have an impact in patient’s prognosis in a variety of solid tumors, including head–neck carcinomas [11, 12, 16, 19–21].

In the present study, we examined how conditions that prevail in tumor microenvironment affect TIL density and TLS formation. Authors have proposed different ways for the evaluation of TILs [22, 23]. In 2014, Salgado et al. proposed the “First International Guidelines on TIL-assessment in breast cancer” by the International TILs Working Group (ITWG). Briefly, they estimated the lymphocytic infiltration as the percentage of stromal TILs in the whole area of stroma tissue, when artifacts, necrotic areas, and polymorphonuclear cells were ruled out of evaluation [24]. Hendry et al., in 2017, modified the Guidelines published by ITWG and took the methodology a little further, so as to be used for solid tumors in general, and not only for breast carcinomas [25]. In our study, we calculated the number of lymphocytes in the invading front of the tumor and inside the tumor stroma in × 400 magnification in all available optical fields, and the total number of lymphocytes was divided by the total amount of high-power optical fields to score each case. According to the median value, cases were categorized as high- and low-TIL density. This method, although laborious, overcomes the subjectivity of each pathologist in the assessment of lymphocytic infiltration. It also makes possible the comparison of TIL density among cases with different sizes of tumor sections.

Using a similar approach to quantify TLSs density, we counted the number of all TLSs in the whole slide at × 400 magnification and then divided it with the total number of optical fields. Tumors were categorized as low- and high-TLS density. As TLS, we considered a relatively circumscribed accumulation of lymphocytes, whether it showed a germinal center or not. Some authors use immunohistochemistry for the assessment of TLSs, but standard markers are not yet proposed for different solid tumors [26–28].

TIL density and TLS density in the invasive tumor front and in the inner stroma of the tumor showed no statistically significant correlation with tumor size in our study, a fact that comes in agreement with Brand et al., who also found no association between TILs and tumor size in melanomas in samples of mice [29]. On the other hand, Sha et al. and Zhang et al. reported that low-TIL density was related to larger size of tumor in gastric adenocarcinomas [30, 31]. Concerning the ability of laryngeal cancer to invade into the organ, we showed a relation among low levels of TILs inside the tumor stroma and invasion of cartilaginous structures. Other studies in oral, gastric, bladder cancer, or melanoma have shown a similar association of low-TIL density with tumor invasiveness [32–35].

In our series of laryngeal SCCs, poor presence of TILs in the invading front of the tumor was significantly related with high metastatic lymph node involvement, which is also supported by a variety of authors in their studies about different types of cancers [12, 30–32, 34]. Regarding TLSs, some reports suggest a connection of high TLS presence with lower N-stage [36, 37]. The growth ability of laryngeal SCC, as indicated by proliferative index MIB1, was also directly associated with poor TIL density in the invading tumor front (*p* = 0.006) and in inner tumor areas. On the contrary, Kramer et al. and Zadka. et al. noted a positive correlation between brisk inflammation and MIB1 expression in basal cell carcinoma and colorectal cancer, respectively [38, 39], and Zhang et al. related TLS presence with MIB1 [28]. Lastly, laryngeal tumors in this study showed a reactivity in a very low percentage of cancer cells (0–4%), probably because of a non-HPV etiology.

Tumor budding remains on the frontline of research, with some pathologist using this prognostic indicator even in daily practice in their pathology reports. Our statistical analysis strongly associated high TB numbers with low-TIL density in peripheral and inner tumor areas. Safaa et al. and Zhan et al. reached to the same results, as they established a negative correlation between tumor budding and TIL density [40, 41], although Lang-Schwarz et al. found no correlation among them in a series of colorectal carcinomas [42]. The presence of CAFs was assessed with the use of SMA immunohistochemistry. We report no significant difference of SMA expression in regard to TIL and TLS densities, while some authors suggest a negative association of dense fibrosis/SMA expression with TIL levels [39, 43].

TIL and TLS densities were significantly lower in inner tumor areas compared to the invading tumor front, which suggests poor survival ability of TILs in the adverse microenvironmental conditions prevailing away from the tumor periphery. The fact that FOXP3^+^ TILs, in contrast to CD8^+^ and CD4^+^ TILs, did not decrease in inner tumor areas further suggests that regulatory T-cells may better survive in the microenvironment of the tumor body. As hypoxia and acidity are the main physicochemical conditions in tumors, we examined the expression of proteins that drive such conditions. Hypoxic microenvironmental conditions, as reflected by high expression of HIF1α were linked with poor TIL density in the tumor-invading front and in inner tumor areas. These results were in agreement with reports by Giatromanolaki et al. and Koukourakis et al. in a series of breast and head/neck carcinomas, respectively [12, 16]. Additionally, high HIF1α was linked with poor TLS density in the tumor-invading front and in inner tumor areas. Furthermore, Giatromanolaki et al. supported an association of high-TIL density in the invading front of the tumor with low LDH5 expression in breast cancer [16], while, in the current study, we further suggest a relation between high expression of LDH5 and poor TLS density in the tumor-invading front and in inner tumor areas.

Moreover, as regards vascular density in laryngeal SCC, the analysis showed that VD in the invading tumor front was directly related to TIL density (*p* = 0.02) and TLS density (*p* = 0.04). Such TILs were, however, of regulatory type, as FOXP3^+^ lymphocytes and high FOXP3/CD8 ratio characterizes tumors with high VSA ability. As VEGF is a major vascular survival factor, high VEGF expression could be the cause of the above correlation. VEGF is also a major factor driving regulatory pathways of T-cell differentiation [44, 45].

The advantage of this study was that whole specimens of the larynx were examined instead of the biopsy tissue. An extensive statistical analysis was performed, and an abundance of information was obtained in a single report, exploring in depth the interaction of both histopathological parameters and tumoral microenvironmental conditions with immune response. A disadvantage of the study is the lack of data regarding the postoperative prognosis of patients. Nevertheless, in a recent study on bioptic material from laryngeal SCC patients treated with chemo-RT, we showed that low-TIL density was strongly linked with poor survival [12]**.** The current study's plethora of information about the impact of tumor histological characteristics and microenvironment on immune response could be of use for further research and potential interventions to boost immunity against cancer cells and prevent spread and metastasis.

## Conclusions

In the current study, we showed the decisive interaction of immune response against cancer cells with the prevailing microenvironmental conditions. Poor lymphocytic infiltration and low-TLS density were related with hypoxic background (higher HIF1a and LDH5 expression), metastasis to locoregional lymph nodes, a higher number of tumor buds in the invading front, and enhanced angiogenetic ability of the tumor. Among different TIL subtypes, CD4^+^, FOXP3^+^ TILs, and FOXP3^+^/CD8^+^ ratio were directly associated with vascular survival ability. The above results could be used for possible interventions to block immunosuppressive interactions with immune cells and, thus, boost immunity against cancer cells.

## Supplementary Information

Below is the link to the electronic supplementary material.

Supplementary file1 (DOCX 19 KB)

Supplementary file2 Supplemental Figure 1s Schematic representation of the x40 optical fields applied in the invading tumor front (t1) and inner tumor areas (t2,t3) to score TILs, TLS, Vascular Density and other variables analyzed in the current study (TIF 3600 KB)

## Data Availability

All histopathological and clinical data are available in the Department of Pathology of the Democritus University of Thrace.

## Declarations

### Research involving Human and Animal Participants

Authors declare that the conduct of the study complied with the Ethical standards demanded for research on human tissue samples.
