# Supplementary material for: Cancer Microenvironment Defines Tumor-Infiltrating Lymphocyte Density and Tertiary Lymphoid Structure Formation in Laryngeal Cancer
Source: Head Neck Pathol. 2022 Dec 31;17(2):422–32. doi: 10.1007/s12105-022-01517-7 (PMC10293152; doi:10.1007/s12105-022-01517-7)
Supplement: Supplementary file 1 — Supplementary file1 (DOCX 19 KB) [file 12105_2022_1517_MOESM1_ESM.docx]

**Supplemental Table 1s**

Linear regression analysis results of T-cell subtypes vs. % of cancer cell expressing HIF1α, LDH5, vascular density in the invading front (t1) and inner areas (t2,t3) and the vascular survival ability (assessed as VSA t2 and VSA t3).

| **INVADING FRONT** | |  |  |  |  |  |  |  |  |
| --- | --- | --- | --- | --- | --- | --- | --- | --- | --- |
|  | HIF1α (c) | HIF1α (n) | LDH5 (c) | LDH5 (n) | VD t1 | VD t2 | VD t3 | VSA t2 | VSA t3 |
| **CD8** |  |  |  |  |  |  |  |  |  |
| **R square** | 0.01 | 0.00 | 0.06 | 0.28 | 0.00 | 0.02 | 0.07 | 0.01 | 0.03 |
| **P value** | 0.61 | 0.84 | 0.69 | 0.09 | 0.71 | 0.43 | 0.14 | 0.62 | 0.36 |
| **CD4** |  |  |  |  |  |  |  |  |  |
| **R square** | 0.02 | 0.07 | 0.14 | 0.14 | 0.02 | 0.00 | 0.01 | 0.05 | 0.00 |
| **P value** | 0.42 | 0.14 | 0.32 | 0.34 | 0.40 | 0.75 | 0.59 | 0.22 | 0.82 |
| **FOXP3** |  |  |  |  |  |  |  |  |  |
| **R square** | 0.07 | 0.03 | 0.04 | 0.17 | 0.00 | 0.03 | 0.02 | **0.12** | 0.01 |
| **P value** | 0.12 | 0.35 | 0.79 | 0.28 | 0.80 | 0.31 | 0.46 | **0.05** | 0.61 |
| **CD4/CD8** |  |  |  |  |  |  |  |  |  |
| **R square** | 0.02 | 0.02 | 0.14 | 0.05 | 0.02 | 0.00 | 0.06 | 0.00 | 0.01 |
| **P value** | 0.40 | 0.47 | 0.36 | 0.72 | 0.45 | 0.72 | 0.17 | 0.80 | 0.55 |
| **FOXP3/CD8** |  |  |  |  |  |  |  |  |  |
| **R square** | 0.06 | 0.04 | 0.001 | 0.07 | 0.01 | 0.00 | 0.00 | 0.04 | 0.00 |
| **P value** | 0.16 | 0.29 | 0.95 | 0.64 | 0.56 | 0.86 | 0.90 | 0.28 | 0.98 |

| **INNER AREAS** |  |  |  |  |  |  |  |  |  |
| --- | --- | --- | --- | --- | --- | --- | --- | --- | --- |
|  | HIF1α (c) | HIF1α (n) | LDH5 (c) | LDH5 (n) | VD t1 | VD t2 | VD t3 | VSA t2 | VSA t3 |
| **CD8** |  |  |  |  |  |  |  |  |  |
| **R square** | 0.01 | 0.01 | 0.24 | 0.01 | 0.01 | 0.01 | 0.02 | 0.01 | 0.03 |
| **P value** | 0.51 | 0.64 | 0.16 | 0.87 | 0.68 | 0.68 | 0.47 | 0.54 | 0.38 |
| **CD4** |  |  |  |  |  |  |  |  |  |
| **R square** | 0.00 | 0.06 | 0.005 | 0.005 | 0.00 | 0.13 | 0.03 | **0.20** | 0.04 |
| **P value** | 0.90 | 0.16 | 0.91 | 0.74 | 0.97 | 0.04 | 0.30 | **0.008** | 0.29 |
| **FOXP3** |  |  |  |  |  |  |  |  |  |
| **R square** | 0.04 | 0.03 | 0.14 | 0.005 | 0.04 | 0.01 | 0.02 | **0.15** | 0.08 |
| **P value** | 0.28 | 0.31 | 0.42 | 0.75 | 0.27 | 0.57 | 0.40 | **0.02** | 0.12 |
| **Cd4/CD8** |  |  |  |  |  |  |  |  |  |
| **R square** | 0.01 | 0.01 | 0.20 | 0.07 | 0.01 | 0.00 | 0.00 | 0.00 | 0.01 |
| **P value** | 0.60 | 0.51 | 0.24 | 0.65 | 0.67 | 0.83 | 0.85 | 0.90 | 0.67 |
| **FOXP3/CD8** |  |  |  |  |  |  |  |  |  |
| **R square** | 0.02 | 0.01 | 0.14 | 0.03 | 0.02 | 0.01 | 0.00 | **0.11** | 0.02 |
| **P value** | 0.45 | 0.65 | 0.42 | 0.85 | 0.44 | 0.58 | 0.74 | **0.05** | 0.44 |
