# Supplementary figures and images for: Cancer Microenvironment Defines Tumor-Infiltrating Lymphocyte Density and Tertiary Lymphoid Structure Formation in Laryngeal Cancer
Source: Head Neck Pathol. 2022 Dec 31;17(2):422–32. doi: 10.1007/s12105-022-01517-7 (PMC10293152; doi:10.1007/s12105-022-01517-7)

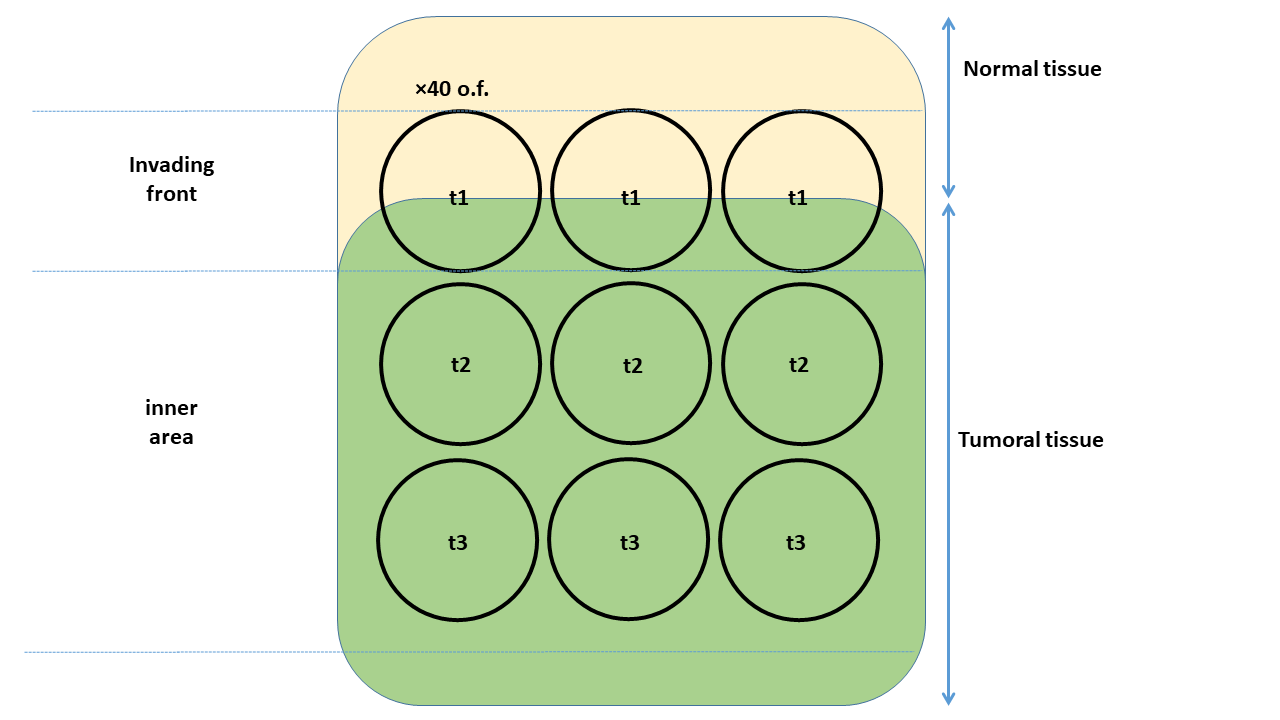

Supplement: Supplementary file 2 — Supplementary file2 Supplemental Figure 1s Schematic representation of the x40 optical fields applied in the invading tumor front (t1) and inner tumor areas (t2,t3) to score TILs, TLS, Vascular Density and other variables analyzed in the current study (TIF 3600 KB) [file 12105_2022_1517_MOESM2_ESM.tif]
